# Comparison Between Human and Rodent Neurons for Persistent Activity Performance: A Biologically Plausible Computational Investigation

**DOI:** 10.3389/fnsys.2021.628839

**Published:** 2021-09-09

**Authors:** Qian Zhang, Yi Zeng, Tielin Zhang, Taoyi Yang

**Affiliations:** ^1^Institute of Automation, Chinese Academy of Sciences (CAS), Beijing, China; ^2^University of Chinese Academy of Sciences, Beijing, China; ^3^Center for Excellence in Brain Science and Intelligence Technology, Chinese Academy of Sciences (CAS), Shanghai, China; ^4^National Laboratory of Pattern Recognition, Institute of Automation, Chinese Academy of Sciences (CAS), Beijing, China

**Keywords:** human neuron, interneuron, neocortex, persistent activity, computational model

## Abstract

Elucidating the multi-scale detailed differences between the human brain and other brains will help shed light on what makes us unique as a species. Computational models help link biochemical and anatomical properties to cognitive functions and predict key properties of the cortex. Here, we present a detailed human neocortex network, with all human neuron parameters derived from the newest Allen Brain human brain cell database. Compared with that of rodents, the human neural network maintains more complete and accurate information under the same graphic input. Unique membrane properties in human neocortical neurons enhance the human brain’s capacity for signal processing.

## Introduction

The neocortex processes higher-level cognitive function, including working memory, attention, and perception (Miller, 2000; Koechlin et al., 2003; Nieder and Miller, 2003; Wood and Grafman, 2003; Bishop et al., 2004). In addition, deep introspection and abstract reasoning are thought to be human-specific (Kandel et al., 2000; Eyal et al., 2016). Different levels of the cortical system are accessible by different methods. As a result of developments in neuroimaging and neurophysiology, we can explore the human neocortex from the macro connectome level (Klein et al., 2010; Ford and Kensinger, 2014; Samara et al., 2017) and the micro cellular level (Beaulieu-Laroche et al., 2018; Colangelo et al., 2019; Gidon et al., 2020). These technologies also allow us to compare features across species, in terms of their function and structure. Determining the differences between human neurons and other species appears to be particularly important because they are considered to play a key role in human high-level cognition and evolution (Golgi, 1906; Cajal et al., 1995; Defelipe, 2011; Mohan et al., 2015).

In this study, we modified the model of a detailed data-driven single prefrontal cortex (PFC) proposed by Hass et al. (2016) (Accession:189160)^1^ and used a different adaptive exponential integrate-and-fire (aEIF) neuron model. The sixth layer has been added to our column model. Sources of anatomical structure include ferret, rodent, and primate PFC experiments (Gao et al., 2003; González-Burgos et al., 2005; Otsuka and Kawaguchi, 2009). Therefore, this model is a neocortical network based entirely on biological experimental data. We used the rodent network for the information maintenance accuracy test, which is reflected by the persistent activity.

Next, we replaced the rodent neuron model with the human neuron model. We analyzed the effect of neuron membrane parameters that differed significantly from rodent brains on network performance, which is the result of integrating many neuron parameters. A single change in the parameters did not yield satisfactory results. By introducing salt and pepper noise into the task, the anti-noise performance of the human neural network exceeds that of the rodent network. We hope to provide a computational framework for predictive modeling to study human-specific cognitive functions.

## Materials and Methods

### Neuron Model

For the adaptive exponential integrate-and-fire (aEIF) model, the voltage *V* and the adaptation variable *w* were expressed using the following two-dimensional differential equations:

(1) $$\text{C}\frac{\text{dV}}{\text{dt}}=-{\text{g}}_{\text{L}}\left(V-{\text{E}}_{\text{L}}\right)+{\text{g}}_{\text{L}}\exp\left(\frac{V-{\text{V}}_{\text{th}}}{\Delta T}\right)+\text{I}-\text{w}$$

(2) $$\tau_{W}\frac{\text{dw}}{\text{dt}}=\text{a}\left(V-{\text{E}}_{\text{L}}\right)-\text{w}$$

If V > V_th_, V → V_r_, w → w + b where *C* is the membrane capacitance, *g*_*L*_ is the leak conductance, *E*_*L*_ is the leak reversal potential, *V*_*r*_ is the reset potential, *V*_*th*_ is the spike threshold, Δ*T* is the slope factor, *I* is the background currents, τ_*w*_ is the adaptation time constant, *a* is the subthreshold adaptation, and *b* is the spike-triggered adaptation.

The rodent neuron parameters in this network were derived from the experimental literature related to PFC and other areas of the neocortex (Markram et al., 2004; Jiang et al., 2015; Mohan et al., 2015; Hass et al., 2016). The human neuron model parameters can be retrieved from the database by following its instruction.^2^

Neuron parameters are presented in Supplementary Tables 1, 2.

### Synaptic Properties

Neurons were connected through three types of synapses (AMPA, GABAA, and NMDA):

(3) $${\text{I}}_{\text{X}}={\text{g}}_{\text{X}}^{\text{max}}\text{s}\left(\text{V}\right)\sum_{{\text{t}}_{\text{sp}}}\text{a}\left({\text{t}}_{\text{sp}}\right)\left({\text{e}}^{-\frac{t-{\text{t}}_{\text{sp}}-\tau_{\text{D}}}{\tau_{\text{off}}^{\text{X}}}}-{\text{e}}^{-\frac{t-{\text{t}}_{\text{sp}}-\tau_{\text{D}}}{\tau_{\text{on}}^{\text{X}}}}\right)\left(V-{\text{E}}_{\text{rev}}^{\text{X}}\right)$$

(4) $$\mathrm{with}s\left(\text{V}\right)=\left\{ \begin{array}{c} 1.08{\left(1+0.19\exp\left(-0.064V\right)\right)}^{-1}\mathrm{for}X=\text{NMDA}\\ 1\mathrm{otherwise} \end{array}\right.$$

$$\text{X}\in \left\{\mathrm{AMPA},{\text{GABA}}_{\text{A}},\mathrm{NMDA}\right\}$$

*E*_*rev*_ is the reversal potential, τ_*off*_ and τ_*on*_ are the onset and offset time constants, *g*_*max*_ is the peak conductance, and τ_*D*_ is the transmission delay. The synapse parameters are presented in Supplementary Tables 4, 5.

Synapses were also equipped with short-term synaptic plasticity (STP) dynamics implemented in the Tsodyks and Markram model (Markram et al., 1998).

(5) $${\text{a}}_{\text{n}}={\text{u}}_{\text{n}}{\text{R}}_{\text{n}}$$

(6) $${\text{u}}_{\text{n}+1}={\text{u}}_{\text{n}}\exp\left(\frac{-\Delta \text{t}}{\tau_{\text{facil}}}\right)+\text{U}\left(1-{\text{u}}_{\text{n}}\exp\left(\frac{-\Delta \text{t}}{\tau_{\text{facil}}}\right)\right)$$

(7) $${\text{R}}_{\text{n}+1}={\text{R}}_{\text{n}}\left(1-{\text{u}}_{\text{n}+1}\right)\exp\left(\frac{-\Delta \text{t}}{\tau_{\text{rec}}}\right)+1-\Delta \exp\left(\frac{-\Delta \text{t}}{\tau_{\text{rec}}}\right)$$

(8) $${\text{R}}_{1}=1-\text{U}$$

where *a*_*n*_ is the relative efficiency, *u*_*n*_ is the utilization of synaptic efficacy, with initial condition s *u*_1_ = *U* and *R*_1_ = 1. τ_*rec*_ is the recovery from depression over time, τ_*facil*_ is the facilitation dominant on time. The value of *U* was 0.25; τ_*facil*_ and τ_*rec*_ were 500 and 300 ms, respectively.

### Neural Network Model

**FIGURE 1:**
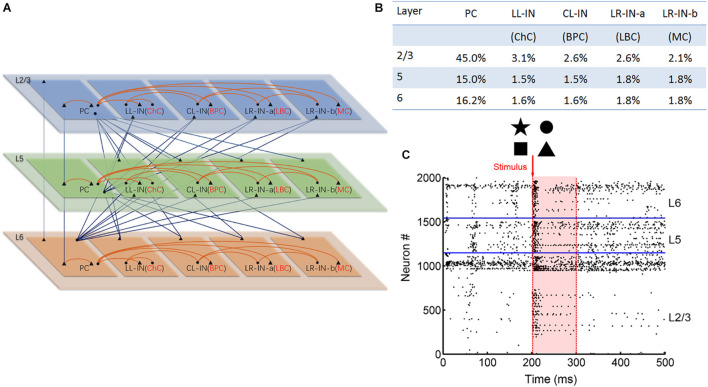
Anatomical and stimulation diagram. **(A)** Laminar connection of a single column. Triangles indicate excitatory synaptic projection and dots indicate inhibitory synaptic projection. The red lines indicate local layer connections, and the blue lines indicate the cross-layer connections (PC, pyramid cell; LL-IN, local-layer connection interneuron; CL-IN, cross-layer connection interneuron; LR-IN, long-range connection interneuron; ChC, chandelier cell; BPC, bipolar cell; LBC, large basket cell; MC, Martinotti cell). **(B)** Parameters of different types of neurons are proportionally distributed across layers. **(C)** Network stimulation diagram. Red arrow shows that different pictures stimulate the L2/3 pyramidal cell. The shades of light red indicate the information maintenance.

The 2,000 neurons were assumed to be organized in a single column and divided into supragranular layers 2/3 (L2/3) and infragranular layer 5 (L5) and layer 6 (L6) (see Figure 1A). The neurons were divided into five subtypes, including: pyramidal cells (PCs) and local-layer connection interneurons (LL-INs) with projections within the same layer. Cross-layer connection interneurons (CL-INs) include bipolar cells (BPCs), which have vertically extended axonal clusters largely within a column (Jiang et al., 2015), and long-range connection interneurons (LR-INs), including large basket cells (LBCs) as LR-IN-a and Martinotti cells (MC) as LR-IN-b (Markram et al., 2004). These LBCs and MCs have large clusters of axons that extend not only across the layers but also across multiple columns. The LBCs have electrophysiological properties similar to those of PCs, meaning that their neuronal parameters are the same as those of PCs in the respective layers (Chrysanthidis et al., 2019).

Neurons were distributed over the five cell types in each layer based on estimates from the literature (Markram et al., 2004). The PCs and interneurons were proportionally distributed (Figure 1B; Beaulieu, 1993; Defelipe, 2011) and randomly connected to different connection probabilities for each pair of cell types based on previous studies (Gibson et al., 1999; Gao et al., 2003; Hass et al., 2016; Supplementary Table 5). All neurons received background currents, which represent synaptic connections from outside the network, both within and outside the column. The excitatory neuron background current was 250 pA, and the interneuron background current was 200 pA.

### Stimulation Paradigm

We converted the binary input image into a 30 × 30 matrix of 0 and 1 s, where 0 represents no spike input and 1 represents the presence of pulse current input. This matrix corresponded to 900 PCs in L2/3. At 201 ms, the corresponding 50 Hz Poisson distributed current pulses were applied to the L2/3 PCs. In the next 99 ms, the 900 L2/3 PCs spiking was recorded (Figure 1C). Zero represented no spiking activity. When spiking occurred, it is represented by 1. The firing of the No: 1-900 PC was transformed into a 1 × 900 (0, 1) vector. Next, this vector is converted into a 30 × 30 matrix. Finally, the (0, 1) matrix was converted into a binary image (0 means white, 1 means black) for output. Noise interference was achieved by adding salt and pepper noise to the input image, as shown in Figure 7A. Every experiment was repeated a total of nine times.

The spike density of the network in the specified time interval was calculated using the following equation:

(9) $${\text{D}}_{\text{spike}}=\frac{{\text{N}}_{\text{spike}}}{{\text{T}}_{\mathrm{time}\mathrm{interval}}}\left(\mathrm{spikes}/\mathrm{ms}\right)$$

where *N*_*spike*_ is the number of spikes in the entire network during the specified time interval, and *T*_*time interval*_ = 100 ms.

The ratio of excited neurons was calculated using the following equation:

(10) $${\text{R}}_{\mathrm{excited}\mathrm{neuron}}=\frac{{\text{N}}_{\mathrm{excited}\mathrm{neuron}}}{{\text{N}}_{\mathrm{stimulate}\mathrm{neuron}}}\times 100\%$$

where *N*_*excited neuron*_ is the number of neurons in excited state. An excited neuron is defined as a 50% increase in spike density after the neuron receives a current stimulation *N*_*stimulate neuron*_ is the number of neurons receiving current stimulation.

The signal transfer accuracy was calculated using the following equation:

(11) $$p_{accuracy}=\frac{N_{input=output\left(L2/3PC\right)}}{N_{L2/3PC}}\times 100\%$$

where *N*_*input* = *output* (L2/3 PC)_ is the number of neurons in the L2/3 PC layer in which the input and output are consistent: (1) the neuron receives pulse current (201 ms), and the neuron emits action potentials (202-300 ms). (2) In the stimulation phase, the neuron does not receive pulse current (201 ms), and the neuron does not emit an action potential (202-300 ms). *N*_*L2/*3P*C*_ is the number of L2/3 PCs.

### Statistical Information

The analysis of variance (ANOVA) was used to assess statistical differences. *P* > 0.05 were considered not significant (ns); ^∗^*P* < 0.05, ^∗∗^*P* < 0.01 and ^∗∗∗^*P* < 0.0001 were considered statistically significant.

### Simulation Details

All simulations were performed in MATLAB. The time step of the neuron models, spikes, synaptic, external events, and network have a maximum time step of 0.05 ms. The resolution displayed by the experimental results is 1 ms. Neurons were initialized with V^*i*^(0) = *E*^*i*^_*L*_, w^*i*^(0) = 0 for all neurons.

## Results

### Key Features of Human Neuron Model Parameters

The rodent neuron model key parameters [membrane capacity (C), leak conductance (g_*L*_), leak reversal potential (E_*L*_), reset potential (V_*r*_), and threshold potential (V_*th*_)] were estimated from the experimental literature (Koester and Johnston, 2005; Hass et al., 2016). Previous researchers obtained neuron parameters for a large number of in vitro recordings from different cell types from the PFC of rats and mice. The human neuron model parameters (C, g_*L*_, E_*L*_, and V_*th*_) were derived from the latest Allen Brain human brain cell database [Allen Cell Types Database (2015)]^3^ (Hawrylycz et al., 2012), which is open access. The first slew of human data includes the electrical properties of 300 different types of neurons from 36 people. According to dendrite type, these are only divided into spiny, aspiny, and sparse spiny types. Excitatory neurons have spines, and inhibitory neurons are typical none or very sparsely spiny (White, 1989; Juorio, 1998; Markram et al., 2004). Therefore, our human neuron model used spiny neurons as PCs and aspiny neurons as LL-INs. To clearly reflect these differences, we used the parameters of the human neuron to subtract the parameters of the rodent neuron and listed the percentage differences in Supplementary Table 3 and Supplementary Figure 1. There were other membrane parameters that had distinctive values, especially in C (L5 PC and L6 PC) and V_*th*_ (L2/3 LL-IN, L5 LL-IN, and L6 LL-IN). The C of human L5 and 6 PCs was nearly half that of rodent neurons. We and Eyal et al. (2016) both found that the membrane capacitance of certain types of human PCs in the neocortex was significantly lower than that of rodents. A lower C enhances both synaptic charge-transfer from dendrites to soma and spike propagation along the axon. In addition, both V_*r*_ and V_*th*_ were higher in human neurons than in rodent neurons.

At the single neuron level, we used the aEIF model (Brette and Gerstner, 2005), which has been shown to reproduce rich firing patterns (Naud et al., 2008). The different voltage responses of the aEIF model to a short square current are shown in Supplementary Figures 2A–F. Different types of neurons exhibited their own regular spiking waveforms under the same stimulus. Two thousand neurons were layered to form a functional column (Figure 1A, see the “Materials and Methods” sections for the neural network model). The connections between neurons were very diverse, including feedforward, feedback, and self-connections. Excitatory neurons and interneurons were connected by conductance-based synapses (AMPA, GABA_*A*_, and NMDA) with different connection probabilities according to a previous study (Gibson et al., 1999; Markram et al., 2004; Koester and Johnston, 2005; Frick et al., 2008; Otsuka and Kawaguchi, 2009; Fino and Yuste, 2011; Potjans and Diesmann, 2014) (see section “Materials and Methods” and Supplementary Tables 4, 5 for synapse model). The “Materials and Methods” section provides details on image input and output extraction from the network (Figure 1C).

**FIGURE 2:**
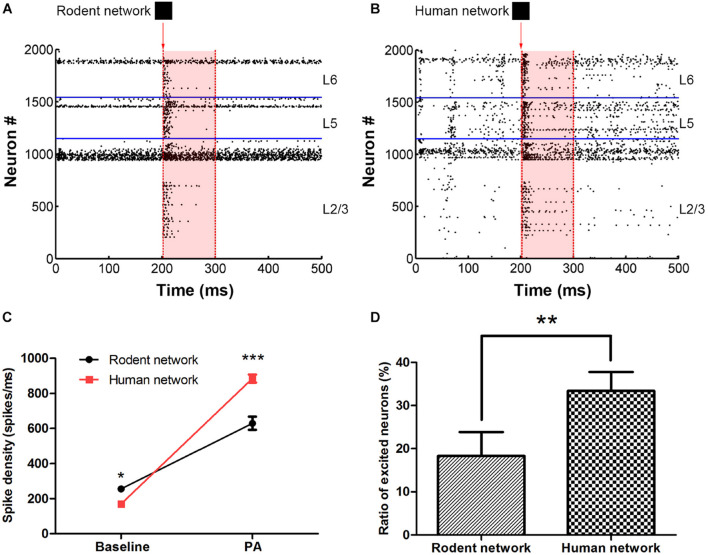
The comparison between rodent **(A)** and human neural network **(B)** under the same image input. The red arrow indicates that in 201 ms, input pattern stimulates the layer 2/3 pyramidal cell. The shades of light red indicate the persistent activity. The horizontal blue line is the dividing line between the layers. **(C)** Statistics representing the network spikes density in the two stages, at baseline and persistent activity (PA). **(D)** The ratio of excited neurons between rodent and human neural network. The error bars represent the standard error of the mean of nine independent experiments, *P < 0.05, **P < 0.01, ***P < 0.0001.

The baseline (100–200 ms) network spike density of the human and rodent were 169.8 ± 2.8 vs. 255.5 ± 6.0 (spikes/ms) (^∗^*P* < 0.05). The persistent activity period (200–300 ms) network spike density of the human and rodent were 884.7 ± 22.8 vs. 629.3 ± 37.6 (spikes/ms) (^∗∗∗^*P* < 0.0001) (Figure 2, see section “Materials and Methods” for the stimulation paradigm and the calculation of the network spike density Eq. 9). At baseline, the spike density of the human neural network was lower than that of the rodent. However, the human network was more active than the rodent network under the same stimulus (Figure 2C). We compared the spiking rate before and after the stimulus (for a 100 ms period) to decide if the neuron really continues to be activated. The ratio of the neurons in an excited state (since the neuron fire rate is more than 50 percent faster after receiving the input, Eq. 10) in the human network was obviously higher than rodent network (33.4 ± 4.4% vs. 18.3 ± 5.5%, ^∗∗^*P* < 0.01) (Figure 2D). The distinctive biophysical properties of neurons in humans resulted in more efficient signal transfer. Human neural networks are less active in their basal state than rodent networks, but firing is easier to maintain after being stimulated.

### Human Neural Network Signal Transfer

**FIGURE 3:**
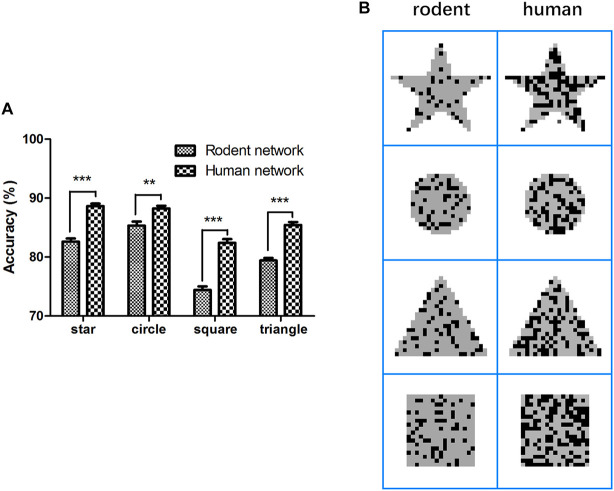
Comparison of accuracy and completeness in rodent and human neural network. **(A)** Network persistent activity performance, **(B)** rodent and human network input (gray) and output (dark) comparison. The error bars represent the standard error of the mean of nine independent experiments, **P < 0.01, ***P < 0.0001.

First, the rodent neuron network L2/3 PC was stimulated by applying four full-field input patterns (star, circle, square, and triangle) within 1 ms. In the next 99 ms, we extracted the output and calculated the accuracy of the signal transfer, as shown in the method representing the stimulation paradigm. The mean accuracy of these four patterns (star, circle, square, and triangle) during the retention of information was 82.6 ± 0.5%, 85.3 ± 0.7%, 74.4 ± 0.4%, and 79.4 ± 0.4%, respectively (Figure 3A, Eq. 11). To determine how neurons affect network performance, we maintained the network structure and other parameters the equally and replaced rodent neuron models with human neuron models, after which we repeated the above experimental paradigm. The mean accuracy of the four patterns (star, circle, square, and triangle) reached 88.6 ± 0.4%, 88.2 ± 0.4%, 82.4 ± 0.6%, and 85.4 ± 0.5%. Compared with the rodent, the human neural network significantly improved the accuracy of output (Figure 3A, ^∗∗^*P* < 0.005) for four different patterns. The accuracy increased by 6.0, 2.9, 8.0, and 6.0%, respectively, and the correct rate of the square image was the highest increase. Notable Figure 3B shows the better performance of the human network in terms of output completeness.

**FIGURE 4:**
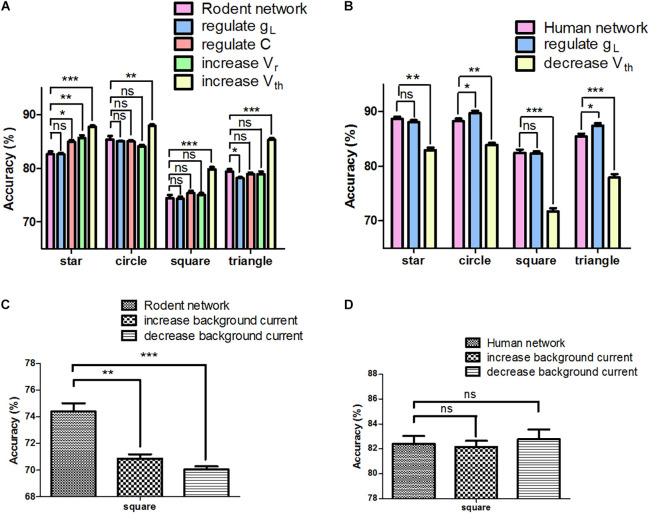
Network signal transfer accuracy after adjusting different parameters individually. **(A)** The performance of rodent neural network after adjusting different parameters. **(B)** The performance of human neural network after adjusting different parameters. **(C)** The rodent neural network performance after adjusting background input. **(D)** The human neural network performance after adjusting background input. The error bars represent the standard error of the mean of nine independent experiments, *P* > 0.05 were considered not significant (ns); *P < 0.05, **P < 0.01, ***P < 0.0001.

In order to further assess the effect of neuron parameters on signal transfer, we adjusted only one parameter from rodent to human, with others remaining invariant at any given time. Supplementary Table 3 shows that for the same parameter, different types of human neurons are not always higher than those of rodents (such as g_*L*_ and C). The most interesting aspect of this graph is that from a computational perspective, not every parameter adjustment contributes to the improvement in accuracy (Figure 4A). For instance, when we individually regulated the g_*L*_, the performance of the network decreased (triangle: 79.4 ± 0.4% vs. 78.2 ± 0.2%, ^∗^*P* < 0.05). Decreasing V_*th*_ had a positive impact on the network performance (square and triangle: 74.4 ± 0.6% vs. 79.8 ± 0.5% and 79.4 ± 0.4% vs. 85.3 ± 0.3%, ^∗∗∗^*P* < 0.0001). Furthermore, we chose the parameters that have a negative (g_*L*_) and positive impact (V_*th*_) on performance in four input images (star, circle, square, and triangle), and adjusted only one parameter from human to rodent at a time in order to keep the other parameters unchanged. Taking the triangle as an example, regulating g_*L*_ had a positive effect on the network (85.5 ± 0.5% vs. 87.4 ± 0.4%, ^∗^*P* < 0.05), and increasing V_*th*_ greatly reduced the performance of the network (85.5 ± 0.5% vs. 77.94 ± 0.6%, ^∗∗∗^*P* < 0.0001) (Figure 4B). It can be inferred as such that the influence of adjusting a single parameter on the network remains unknown, yielding both positive and negative effects. Since adjusting V_*th*_ can improve the accuracy of the rodent network, we tried to increase (PC 300 mA, IN 250 mA) or decrease (PC 200 mA, IN 150 mA) the background current of the rodent network. Interestingly, neither can optimize the performance of the network (Figure 4C). We then adjusted the background current on the human network, and the effect was not significant (Figure 4D, ns *P* > 0.05). The effect achieved by changing global variables was not ideal.

### Effect of Neuron Parameters on the Accuracy

To better analyze the impact of neuron parameters on the network performance in maintaining signals, we performed a non-linear regression fitting on the five types of parameters that were significantly different from rodents (C_*L*5*PC*_, C_*L*6*PC*_, Vth_*L*2/3*LL*–*IN*_, Vth_*L*5*LL*–*IN*_, and Vth_*L*6*LL*–*IN*_). The coefficients of the first and quadratic terms were estimated using iterative least-squares estimation, and the initial value was set to 1. These parameters are all normalized between 0 and 1, and the fitting result was:

(12) $$\begin{array}{c} {\text{P}}_{\text{accuracy}}=71.8+3.3{\text{C}}_{\mathrm{L5PC}}+0.1{\text{C}}_{\mathrm{L6PC}}+1.5{\text{Vth}}_{\mathrm{L2}/3LL\_\mathrm{IN}}+\\ \;1.4{\text{Vth}}_{\mathrm{L5LL}-\mathrm{IN}}+1.7{\text{Vth}}_{\mathrm{L6LL}-\mathrm{IN}}-0.8{\text{C}}_{\mathrm{L5PC}}^{2}\\ \;-1.9{\text{C}}_{\mathrm{L6PC}}^{2}+2.0{\text{Vth}}_{\mathrm{L2}/3LL-\mathrm{IN}}^{2}+1.7{\text{Vth}}_{\mathrm{L5LL}-\mathrm{IN}}^{2}+\\ \;2.2{\text{Vth}}_{\mathrm{L6LL}-\mathrm{IN}}^{2} \end{array}$$

The right side of the equation was combined with similar terms, as follows:

(13) $$\begin{array}{c} {\text{P}}_{\text{accuracy}}=74.3+2.0\left({\text{Vth}}_{\mathrm{L2}/3LL-\mathrm{IN}}^{2}+0.8{\mathrm{Vth}}_{\mathrm{L2}/3LL\_\mathrm{IN}}+1.1\right)\\ \;+1.7\left({\text{Vth}}_{\mathrm{L5LL}-\mathrm{IN}}^{2}+0.8{\text{Vth}}_{\mathrm{L6LL}-\mathrm{IN}}+0.2\right)+\\ \;2.2\left({\mathrm{Vth}}_{\mathrm{L6LL}-\mathrm{IN}}^{2}+0.8{\text{Vth}}_{\mathrm{L6LL}-\mathrm{IN}}+0.2\right)-\\ \;0.8\left({\text{C}}_{\mathrm{L5PC}}^{2}-4.1{\text{C}}_{\mathrm{L5PC}}+1.1\right)-1.9(C_{\mathrm{L6PC}}^{2}-0.1{\text{C}}_{\mathrm{L6PC}}\\ \;+0.1) \end{array}$$

By transformation, we obtained the following equation:

(14) $$\begin{array}{c} {\text{P}}_{\text{accuracy}}=74.3+2.0{\left({\text{Vth}}_{\mathrm{L2}/3LL-\mathrm{IN}}+\frac{1.5}{4.0}\right)}^{2}+\\ \;1.7{\left({\text{Vth}}_{\mathrm{L5LL}-\mathrm{IN}}+\frac{1.4}{3.4}\right)}^{2}+\\ \;2.2{\left({\text{Vth}}_{\mathrm{L6LL}-\mathrm{IN}}+\frac{1.7}{4.4}\right)}^{2}-0.8{\left({\text{C}}_{\mathrm{L5PC}}-\frac{3.3}{1.6}\right)}^{2}-\\ \;1.9{\left({\text{C}}_{\mathrm{L6PC}}-\frac{0.1}{3.8}\right)}^{2} \end{array}$$

**FIGURE 5:**
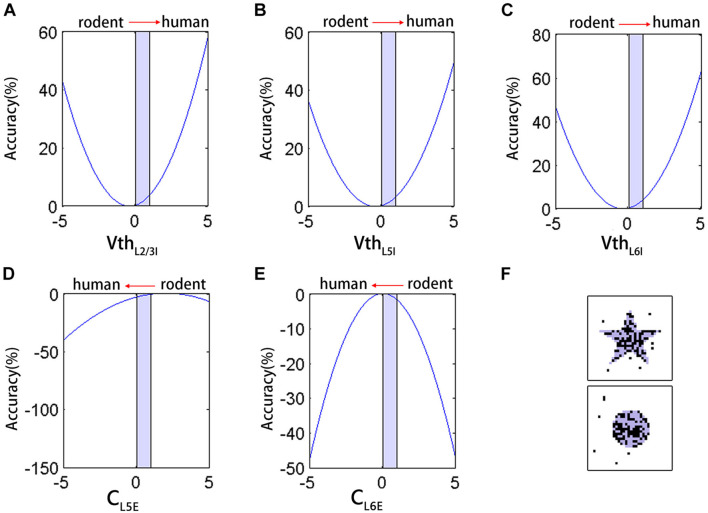
The neuron parameters multiple non-linear regression fitting results. **(A–E)** The effect of Vth_*L*2/3*LL*–*IN*_, V_*thL*5*LL*–*IN*_, Vth_*L*6*LL*–*IN*_, C_*L*5*PC*_, and C_*L*6*PC*_ on the accuracy during [0,1] (light blue shading). Red arrows indicate the direction of the parameters transition from rodent to human. **(F)** The network input (light blue shading) and output (black) after further increase V_*th*_.

According to Eq. 14, the binomial containing each parameter corresponds to a parabola. Based on the fitting results, the increase in V_*thL*2/3*LL*–*IN*_, V_*thL*5*LL*–*IN*_, V_*thL*6*LL*–*IN*_ (Figures 5A–C) and the decrease in C_*L*6*PC*_ (Figure 5E) in [0,1] (red arrows indicate the direction of the parameters transition from rodent to human) all improved the accuracy, except for the decrease in C_*L*5*PC*_ (Figure 5D). The result of this fitting was consistent with the trend of actually adjusting V_*th*_ of the network (Figures 4A,B). The coefficient of C_*L*5*PC*_ was smaller than that of other terms, so the impact on the correct rate is offset. With V_*th*_ as an example, the single change of this parameter had an upper limit to the improvement of the effect. Even if we further increase the V_*th*_ by 25% on human neurons, the correct rate was not improved from a computational point of view, and the output image had a lot of noise (Figure 5F).

### Effect of Synaptic Faster Recovery Time on Accuracy and Anti-noise Performance

**FIGURE 6:**
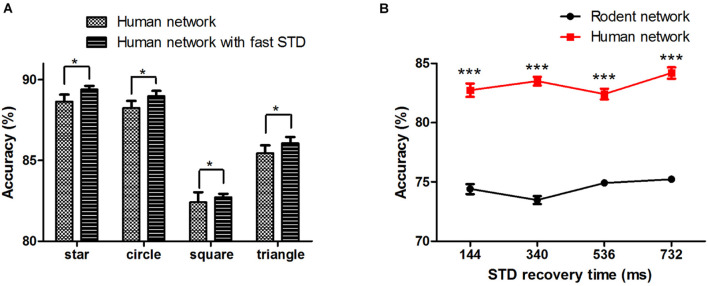
**(A)** Human neural network signal transfer accuracy after adding fast STD recovery. **(B)** The effect of STD recovery time on accuracy, STD: short-term synaptic depression. The error bars represent the standard error of the mean of nine independent experiments, *P < 0.05, ***P < 0.0001.

Synaptic transmission and the plasticity thereof form the building blocks for the processing and storage of information in the brain. Whether human synapses are more efficient at transferring signals between neurons remains to be demonstrated. We added the human short-term synaptic depression (STD) recovery feature to the existing network, which was reflected in the time of recovery from short-term synaptic depression (τ_*rec*_ :144 ± 13 ms with fast STD recovery vs. 536 ± 40 ms without fast STD recovery). The results showed that when we added the characteristics of human STD recovery based on human network, the mean accuracy of the four patterns (star, circle, square, and triangle) reached 89.4 ± 0.2%, 89.0 ± 0.3%, 82.7 ± 0.2%, and 86.1 ± 0.4%, respectively (^∗^*P* < 0.05) (Figure 6A). We then changed and tested the impact of different recovery times on the network (taking the square as an example) (Figure 6B), and we found that there was a non-linear change in performance with changes in STD recovery time.

**FIGURE 7:**
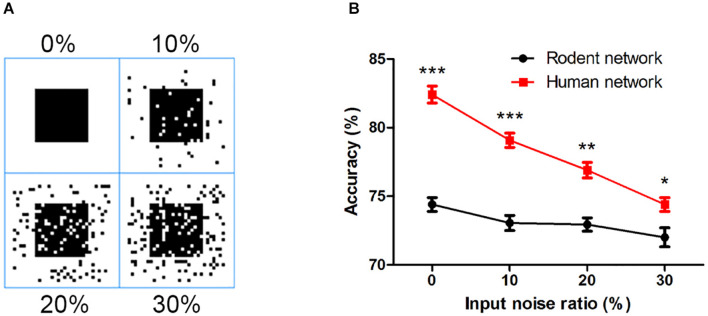
**(A)** The input image with different proportions of salt and pepper noise. **(B)** The influence of salt and pepper noise on accuracy. The error bars represent the standard error of the mean of nine independent experiments, *P < 0.05, **P < 0.01, ***P < 0.0001.

Finally, we examined the network’s ability to filter out noise, a characteristic of robustness in the face of interference. Taking the square as an example, we added different proportions of salt and pepper noise (10, 20, and 30%) to the input image (Figure 7A). As the noise increased, the accuracy of the output of the two networks decreased. Even if the noise increased to 30%, the human network still overall outperformed that of the rodents (Figure 7B), indicating the rodent network is less sensitive to noise than the human network.

## Discussion

Evolution has shaped our brains on multiple levels, from neurons to network connections. Previous work has shown that from the physiological structure the cortex of the human brain is thick, and that human cortical neurons have large and complex dendrites (Mohan et al., 2015; Deitcher et al., 2017), decorated with countless dendritic spines (DeFelipe et al., 2002). From the perspective of electrophysiology, human cortical neurons have unique membrane capacitance and dendrite action potentials (Testa-Silva et al., 2014; Eyal et al., 2016; Gidon et al., 2020). The structure determines the electrical properties, which in turn affects transformations of the synaptic in puts to axonal action potentials as the output. Thus, neurons constitute a key element of the network’s computational power. It is difficult to directly compare the cognitive behaviors of different species based on biological experiments. In contrast, computational modeling presents several clear advantages. This study innovatively seeks to use biologically realistic computational models to compare the performance of brain networks in different species during the same cognitive task. Our simulation results demonstrate the effect of human neurons on improving signal transmission accuracy. Our findings provide insights into the physiological building blocks that constitute the cellular function, which ultimately give rise to the cortical network behavior.

There are many parameters in the neuron. We selected the most important membrane parameters related to the neuron model for analysis and found that the C of human L5 and L6 PC was nearly half that of rodent neurons, but that the C of human LL-IN was twice that of rodent neurons (Supplementary Table 3 and Supplementary Figure 2). Such low C of PC and high C of LL-IN increase the network excitability and facilitate signal transfer (both synaptic and action potential-encoded information). The firing thresholds of human neurons and rodent neurons are different, such that under the same background current, there are differences in baseline activity. The following results confirm our conclusion that the human neocortex shows greater network activity than rodents under the same stimulus, a trend that is completely opposite at baseline (Figure 2C). These results show that the characteristics of human brain neurons make it more conducive to the effective transmission of action potential without changing the network connection. As such, we tried to adjust a single parameter to optimize the performance of the network, but the impact of parameter adjustment on the network was not always positive, such as in the case of g_*L*_ (Figure 4A). The threshold voltage has an obvious improvement effect on the network, so we tried to adjust the excitability of the entire network. However, the effect was not ideal (Figures 4C,D). Then we illustrate the influence of membrane capacitance and threshold voltage on the accuracy of network output by using multiple nonlinear regression models. From the perspective of predicting the direction of evolution, we found that further increasing the threshold has a limited effect on optimizing the network, and more noise will follow. The above results show that human neurons have selected the best combination of parameters, making them more suitable for tasks related to information storage than rodent neurons.

In this model, we used many types of interneurons, across which the division of labor in information maintenance differed (Zhang et al., 2020). The network is composed of numerous neurons, so changing the parameters of neurons will break the original homeostasis of the network (Wu et al., 2020). Although connectivity information is critical to the performance of the network, data on the detailed biological connections of the prefrontal lobe of the human brain remain very limited. At the same time, considering the validity of the comparison, we did not change the connections of the network but only replaced the neuron model. We believe that the characteristics of human neurons also play a leading role in signal transfer and completeness.

Our work has several limitations. One important simplification limiting this study consists in the reduction to layer 4, and the fact that the structure of layer 6 is the same as that of layer 5, which is based on the previous motor cortex study (Weiler et al., 2008). Due to the lack of human PFC column structure connection data, we used rodent PFC connectivity instead. In our study, we did not analyze the synapse model parameters but adopted a unified STP model. We restricted our attention to the neuron membrane parameters. The influence of synaptic parameters of different species on the network is also important and valuable. Without having enough parameters to support, we cannot carry out a comprehensive simulation and comparison. With further research, the human cortex connection data will be increasingly refined. Thereafter, we may have even a deeper and more comprehensive comparison for assessing the uniqueness of the human brain from an evolutionary perspective.

## Data Availability Statement

The original contributions presented in the study are included in the article/Supplementary Material, further inquiries can be directed to the corresponding authors.

## Author Contributions

QZ and YZ designed the experiments and wrote the manuscript. QZ performed the simulation of the experiment. TZ and TY directed the parameter acquisition. All authors reviewed the manuscript.

## Conflict of Interest

The authors declare that the research was conducted in the absence of any commercial or financial relationships that could be construed as a potential conflict of interest.

## Publisher’s Note

All claims expressed in this article are solely those of the authors and do not necessarily represent those of their affiliated organizations, or those of the publisher, the editors and the reviewers. Any product that may be evaluated in this article, or claim that may be made by its manufacturer, is not guaranteed or endorsed by the publisher.

## Footnotes

1
http://senselab.med.yale.edu/ModelDB/

2
http://alleninstitute.github.io/AllenSDK/cell_types.html

3
http://celltypes.brain-map.org/
