# Supplementary material for: Comparison Between Human and Rodent Neurons for Persistent Activity Performance: A Biologically Plausible Computational Investigation
Source: Front Syst Neurosci. 2021 Sep 9;15:628839. doi: 10.3389/fnsys.2021.628839 (PMC8459009; doi:10.3389/fnsys.2021.628839)
Supplement: Supplementary file 1 [file Table_1.DOCX]

Supplementary Material

# Supplementary Figures and Tables

## Supplementary Figures


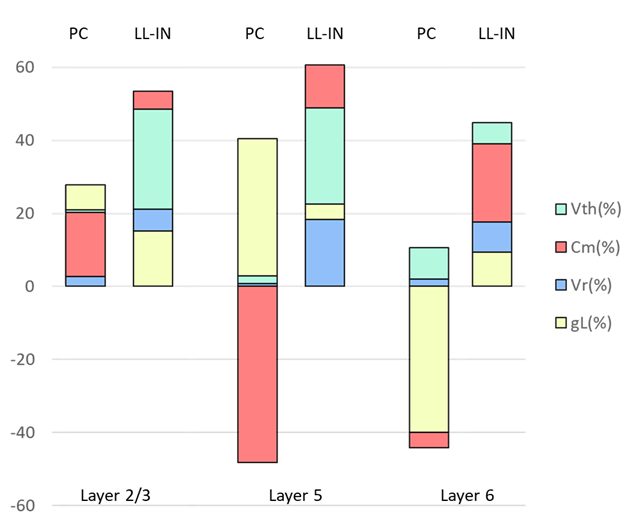


**Supplementary Figure 1.** Neuron parameter comparison. PC stands for the pyramidal cell, LL-IN stands for local layer interneuron. C is the membrane capacitance, g_L_ is the leak conductance，V_r_ is the reset potential, V_th_ is the spike threshold.


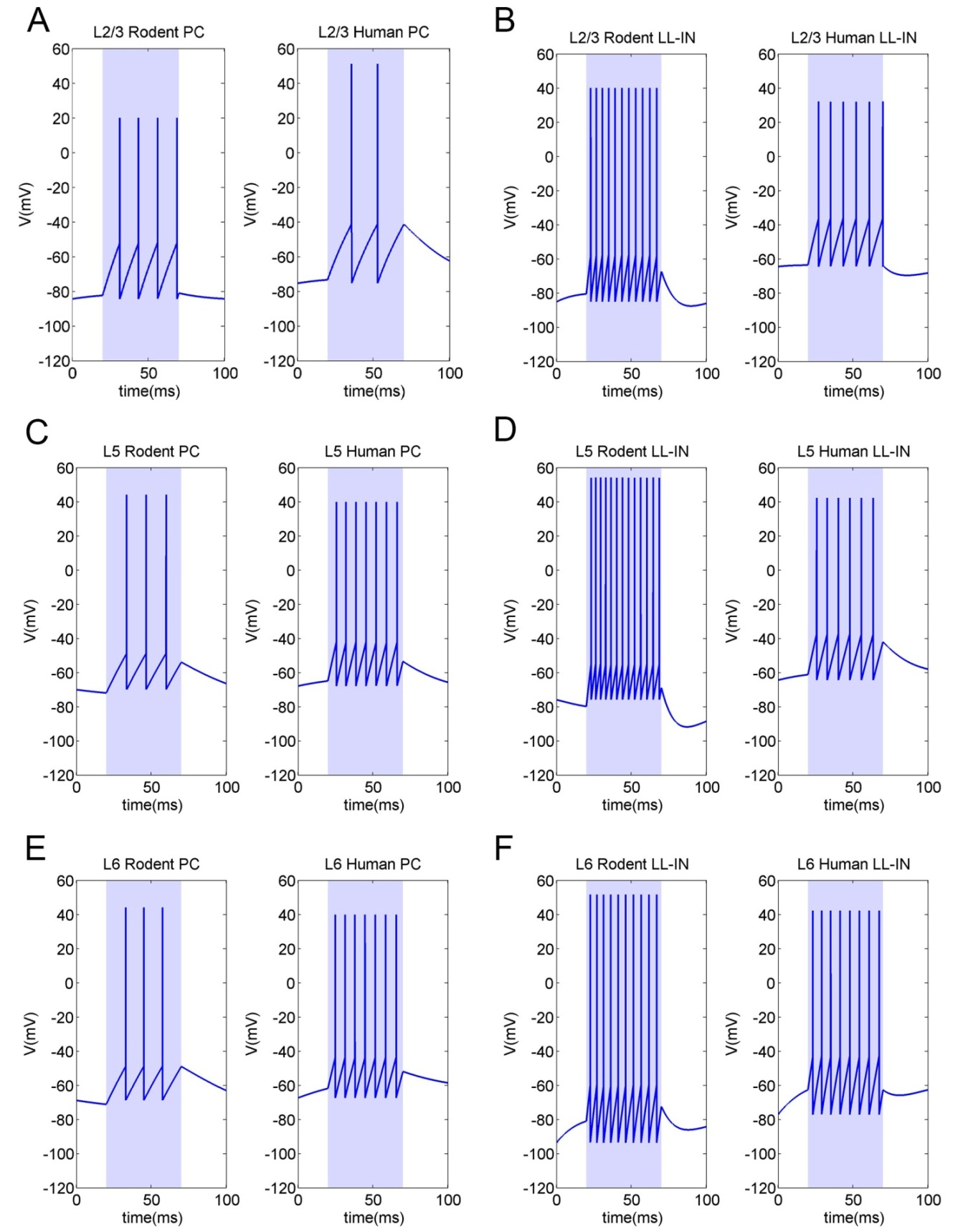


**Supplementary Figure 2.** Neuron voltage curve. The response of different types of neurons under 500pA short-square current stimulation (light blue shading). Pyramid cell (PC), local-layer connection interneuron (LL-IN).

## Supplementary Tables

**Supplementary** **Table** **1.**

Related to neuron parameters.

| Layer | 2/3 | | | | 5 | | | | 6 | | | |
| --- | --- | --- | --- | --- | --- | --- | --- | --- | --- | --- | --- | --- |
| Type | PC | | LL-IN  (ChC) | | PC | | LL-IN  (ChC) | | PC | | LL-IN  (ChC) | |
| Space | rodent | human | rodent | human | rodent | human | rodent | human | rodent | human | rodent | human |
| C(pF) | 165.4  (54.7) | 198.8  (50.9) | 59.4  (9.5) | 91.2  (51.6) | 241.5  (68.0) | 125.0  (13.3) | 53.8  (9.3) | 86.4  (16.2) | 249.2  (64.9) | 139.0  (42.9) | 60.8  (11.4) | 84.5  (11.5) |
| g_L_(nS) | 7.2  (1.6) | 9.2  (1.2) | 5.3  (0.8) | 6.1  (0.9) | 7.4  (1.6) | 4.4  (0.7) | 4.9  (0.8) | 6.0  (0.8) | 7.5  (1.8) | 4.5  (1.6) | 5.3  (0.8) | 5.8  (0.8) |
| E_L_(mV) | -85.0  (4.7) | -75.9  (4.4) | -85.2  (5.0) | -67.7  (4.9) | -80.6  (6.4) | -68  (5.8) | -85.2  (3.8) | -64.1  (7.7) | -80.6  (6.4) | -63  (5.7) | -84.7  (5.1) | -64.7  (4.2) |
| V_r_(mV) | -84.3  (23.9) | -82.0  (26.7) | -95.1  (10.7) | -74.9  (8.7) | -67.8  (12.7) | -67.3  (14.0) | -95.3  (7.6) | -77.8  (14.7) | -68.8  (12.8) | -67.4  (14.4) | -93.6  (8.4) | -77.1  (10.7) |
| V_th_(mV) | -52.1  (4.9) | -41.2  (3.4) | -79.0  (4.3) | -40.6  (8.1) | -48.6  (5.9) | -47.2  (6.2) | -80.4  (2.4) | -41.1  (7.9) | -48.8  (6.5) | -43.6  (6.3) | -78.0  (4.2) | -43.0  (10.1) |
| b(pA) | 7.2  (6.8) | | 34.9  (17.9) | | 8.3  (2.7) | | 34.9  (17.9) | | 8.3  (2.7) | | 34.9  (17.9) | |
| τ_w_ (ms) | 15.2  (2.7) | | 121.8  (41.2) | | 15.2  (2.7) | | 107.8  (64.1) | | 15.2  (2.7) | | 107.8  (64.1) | |
| a(nS) | 2.0 | | | | | | | | | | | |
| Δ_T_(mv) | 20.0 | | | | | | | | | | | |

Mean and standard deviation (in parentheses) of the parameters of the neuron model for the different neuron types used in the network. PC stands for the pyramidal cell, LL-IN stands for local layer interneuron, chandelier cell ( ChC). C is the membrane capacitance, g_L_ is the leak conductance, E_L_ is the leak reversal potential，V_r_ is the reset potential, V_th_ is the spike threshold, b is the spike-triggered adaptation, τ_W_ is the adaptation time constant , a is the subthreshold adaptation and ΔT is the slope factor.

**Supplementary** **Table** **2.**

Related to neuron parameters.

| Layer | 2/3 | | | 5 | | | 6 | | |
| --- | --- | --- | --- | --- | --- | --- | --- | --- | --- |
| Type | CL-IN  (BPC) | LR-IN-a  (LBC) | LR-IN-b  (MC) | CL-IN  (BPC) | LR-IN-a  (LBC) | LR-IN-b  (MC) | CL-IN  (BPC) | LR-IN-a  (LBC) | LR-IN-b  (MC) |
| C(pF) | 10.0  (2.3) | 167.9  (60.5) | 86.3  (30.2) | 83.6  (13.1) | 264.6  (67.2) | 82.9  (22.7) | 82.9  (10.6) | 246.1  (87.1) | 78.6  (29.1) |
| g_L_(nS) | 3.9  (0.4) | 7.2  (1.6) | 2.9  (0.4) | 3.4  (0.5) | 7.5  (1.7) | 3.0  (0.5) | 4.1  (0.4) | 7.2  (1.9) | 3.1  (0.5) |
| E_L_(mV) | -84.9  (4.0) | -84.1  (4.6) | -70.6  (6.1) | -76.8  (6.7) | -82.4  (5.5) | -72.8  (8.0) | -85.2  (3.9) | -80.4  (5.7) | -72.7  (6.4) |
| V_r_(mV) | -77.4  (16.1) | -68.1  (7.9) | -66.3  (8.3) | -92.3  (51.2) | -69.6  (12.6) | -66.6  (8.6) | -94.5  (47.1) | -67.3  (13.3) | -53.4  (7.3) |
| V_th_(mV) | -43.8  (4.9) | -52.1  (5.6) | -42.4  (5.9) | -46.2  (3.3) | -50.3  (6.0) | -48.2  (4.3) | -60.1  (4.4) | -48.1  (4.9) | -46.4  (5.3) |
| b(pA) | 34.9  (3.9) | 6.9  (5.6) | 3.7  (2.9) | 4.1  (3.8) | 6.6  (5.5) | 4.2  (3.6) | 4.4  (3.0) | 5.3  (4.9) | 4.2  (2.8) |
| τ_w_ (ms) | 4.4  (1.2) | 3.9  (0.2) | 9.1  (3.6) | 4.1  (1.4) | 4.9  (0.2) | 9.1  (2.8) | 3.3  (1.7) | 4.9  (0.2) | 10.9  (3.7) |
| a(nS) | 2.0 | | | | | | | | |
| ΔT (mv) | 20.0 | | | | | | | | |

Mean and standard deviation (in parentheses) of the parameters of the neuron model for the different neuron types used in the network. PC stands for the pyramidal cell, LL-IN stands for local layer interneuron, CL-IN stands for cross layer interneuron and LR-IN stands for long range interneuron, local-layer connection interneuron (LL-IN), cross-layer connection interneuron (CL-IN), long-range connection interneuron (LR-IN), bipolar cell (BPC), large basket cell (LBC), and Martinotti cell (MC). C is the membrane capacitance, g_L_ is the leak conductance, E_L_ is the leak reversal potential，V_r_ is the reset potential, V_th_ is the spike threshold, b is the spike-triggered adaptation, τ_w_ is the adaptation time constant , a is the subthreshold adaptation and ΔT is the slope factor.

**Supplementary** **Table** **3.**

Neuron parameter comparison.

| Layer | 2/3 | | 5 | | 6 | |
| --- | --- | --- | --- | --- | --- | --- |
| Type | PC | LL-IN | PC | LL-IN | PC | LL-IN |
| C(%) | 20.2 | 53.5 | -48.2 | 60.6 | -44.2 | 39.0 |
| g_L_(%) | 27.8 | 15.1 | -40.5 | 22.5 | -40.0 | 9.4 |
| V_r_(%) | 2.7 | 21.2 | 0.7 | 18.4 | 2.0 | 17.6 |
| V_th_(%) | 20.9 | 48.6 | 2.9 | 48.9 | 10.7 | 44.9 |

PC stands for the pyramidal cell, LL-IN stands for local layer interneuron. C is the membrane capacitance, g_L_ is the leak conductance,，V_r_ is the reset potential, V_th_ is the spike threshold

**Supplementary** **Table** **4.**

Related to synapses parameters.

|  | GABA_A_ | AMPA | NMDA |
| --- | --- | --- | --- |
| E_rev_(mV) | -70.0 | 0.0 | 0.0 |
| τ_on_(ms) | 3.0 | 1.4 | 4.3 |
| τ_off_(ms) | 40.0 | 10.0 | 75.0 |

E_rev_ is the reversal potential, τ_off_ and τ_on_ are the onset and the offset time constants.

**Supplementary** **Table** **5.**

Related to connection parameters.

| **pre** | **post** | **p_con_** | **g_max_** | **τ_D_(ms)** |
| --- | --- | --- | --- | --- |
| PC L2/3 | PC L2/3 | 0.139 | 0.84 | 1.55 |
| PC L2/3 | PC L5 | 0.233 | 0.95 | 1.91 |
| PC L5 | PC L2/3 | 0.045 | 0.84 | 2.75 |
| PC L5 | PC L5 | 0.081 | 0.88 | 1.56 |
| PC L2/3 | LL-IN L2/3 | 0.325 | 1.34 | 0.96 |
| PC L2/3 | CL-IN L2/3 | 0.159 | 0.47 | 0.96 |
| PC L2/3 | CC-IN L2/3 | 0.325 | 1.34 | 0.96 |
| PC L2/3 | LR-IN L2/3 | 0.290 | 0.25 | 0.96 |
| PC L2/3 | LL-IN L5 | 0.087 | 0.77 | 1.18 |
| PC L2/3 | CL-IN L5 | 0.080 | 0.27 | 1.18 |
| PC L2/3 | CC-IN L5 | 0.087 | 0.77 | 1.18 |
| PC L2/3 | LR-IN L5 | 0.150 | 0.14 | 1.18 |
| PC L5 | LL-IN L2/3 | 0.188 | 1.52 | 1.05 |
| PC L5 | CL-IN L2/3 | 0.092 | 0.53 | 1.05 |
| PC L5 | CC-IN L2/3 | 0.188 | 1.52 | 1.05 |
| PC L5 | LR-IN L2/3 | 0.168 | 0.28 | 1.05 |
| PC L5 | LL-IN L5 | 0.333 | 2.30 | 0.60 |
| PC L5 | CL-IN L5 | 0.080 | 0.13 | 0.60 |
| PC L5 | CC-IN L5 | 0.333 | 2.30 | 0.60 |
| PC L5 | LR-IN L5 | 0.362 | 1.91 | 0.60 |
| LL-IN L2/3 | PC L2/3 | 0.466 | 2.30 | 1.25 |
| CL-IN L2/3 | PC L2/3 | 0.301 | 0.13 | 1.25 |
| CC-IN L2/3 | PC L2/3 | 0.466 | 2.30 | 1.25 |
| LR-IN L2/3 | PC L2/3 | 0.710 | 1.91 | 1.25 |
| LL-IN L2/3 | PC L5 | 0.217 | 1.07 | 1.54 |
| CL-IN L2/3 | PC L5 | 0.140 | 0.06 | 1.54 |
| CC-IN L2/3 | PC L5 | 0.217 | 1.07 | 1.54 |
| LR-IN L2/3 | PC L5 | 0.330 | 0.89 | 1.54 |
| LL-IN L5 | PC L2/3 | 0.039 | 0.1 | 1.44 |
| CL-IN L5 | PC L2/3 | 0.027 | 0.04 | 1.44 |
| CC-IN L5 | PC L2/3 | 0.039 | 0.10 | 1.44 |
| LR-IN L5 | PC L2/3 | 0.040 | 0.07 | 1.44 |
| LL-IN L5 | PC L5 | 0.274 | 0.69 | 0.82 |
| CL-IN L5 | PC L5 | 0.173 | 0.30 | 0.82 |
| CC-IN L5 | PC L5 | 0.274 | 0.69 | 0.82 |
| LR-IN L5 | PC L5 | 0.282 | 0.50 | 0.82 |
| IN L2/3 | IN L2/3 | 0.250 | 1.35 | 1.10 |
| IN L5 | IN L6 | 0.600 | 1.35 | 1.11 |

Mean of the synapses connection between pre- and post-synaptic pair neurons, p_con_ is connection probability, g_max_ is peak conductance, *τ_D_* is transmission delay. Layer 6 neurons have the same parameters as layer 5.
